# Supplementary material for: Application of machine learning approaches to develop predictive models for diabetes and hypertension among Bangladeshi Adults
Source: PLOS Glob Public Health. 2026 Feb 17;6(2):e0004797. doi: 10.1371/journal.pgph.0004797 (PMC12912540; doi:10.1371/journal.pgph.0004797)
Supplement: Attachment — Submitted filename: machine learning-reveiw.docx [file pgph.0004797.s001.docx]

The manuscript addresses an important public health issue using contemporary machine learning (ML) methods on nationally representative data. The study is well-motivated, methods are generally sound, and findings are relevant for policy and prevention. However, several areas require clarification, methodological justification, and improved reporting to meet the journal’s standards.

The tenses in the work are not consistent, kindly make all tenses uniform within the manuscript

**Comments**

The authors applied six ML models but did not clearly justify why these specific algorithms were chosen over others. A brief rationale for each model’s relevance to epidemiological prediction would strengthen the methodological rigor.

While SMOTE and ADASYN were used, the rationale for applying both and their differential impact on model performance is not discussed. The manuscript should briefly explain why these methods were chosen and how they affected model outcomes, especially given the

The models show high sensitivity but very low specificity (e.g., 0.127 for diabetes in XGBoost), indicating poor ability to correctly identify non-cases. The authors should discuss the public health implications of this and suggest ways to improve specificity in future work.

The study would benefit from a comparison with traditional statistical models to contextualize the added value of ML in this setting.

The abstract mentions “low specificity across models” but does not provide values. Consider including key specificity figures for transparency.

BMI cutoffs for overweight/obesity should be specified (e.g., ≥25 kg/m²) for international readability.

The discussion could better highlight how these ML findings translate to actionable public health interventions in Bangladesh

Check consistency in terminology (e.g., “AdaBoost” vs. “ADA Boost” in Table 2).

The tenses in the work are not consistent, kindly make all tenses uniform within the manuscript

Kindly go through the entire work and ensure all grammatical errors are checked. Example w “prevalce of both outcomes” in the discussion should be checked
