# Supplementary material for: Application of machine learning approaches to develop predictive models for diabetes and hypertension among Bangladeshi Adults
Source: PLOS Glob Public Health. 2026 Feb 17;6(2):e0004797. doi: 10.1371/journal.pgph.0004797 (PMC12912540; doi:10.1371/journal.pgph.0004797)
Supplement: Attachment — Submitted filename: response letter.docx [file pgph.0004797.s003.docx]

Gulam Muhammed Al Kibria, MBBS, MSPH, PhD

Johns Hopkins University Bloomberg School of Public Health

615 N Wolfe Street, Baltimore, MD 21205

**Associate Editor**

**PLoS Global Public Health**

June 20, 2023

Dear Editor:

This is to inform you that we have prepared a revised version of the manuscript ‘**Application of Machine Learning Approaches to Develop Predictive Models for Diabetes and Hypertension among Bangladesh Adults’** for your consideration in the *PLoS* *Global Public Health*. We thank you and the reviewers for your time and effort. We have provided itemized responses below

***Reviewer #1:***

This manuscript contributes valuable evidence on body image satisfaction and related psychosocial factors among university students in a low- and middle-income country setting. The topic aligns well with global public health priorities concerning mental health, self-perception, and youth well-being.

Strengths:

Large, multi-institutional sample with strong ethical compliance.

Use of validated measurement instruments.

Rich discussion linking findings to regional and global literature.

Revisions Needed:

Clarify measurement procedures and justify BAS-2 categorization; report mean, SD, and range.

Add reliability statistics for the instruments used.

Reconcile numerical inconsistencies between results text and tables.

Confirm regression diagnostics and check for multicollinearity.

Add a note on referral or support options for participants with high PHQ-4 distress scores.

Deposit anonymized data in a public repository to meet open data standards.

Condense and refocus the discussion for clarity.

Overall Recommendation:
Major Revision — The paper is strong in concept and ethics, but the statistical and reporting details require correction and expansion before publication.

**Response:** We appreciate the reviewers’ efforts; however, the comments provided by Reviewer #1 do not correspond to the content, topic, variables, or methodology of our submitted manuscript. Reviewer #1 refers to BAS-2, PHQ-4, body-image measures, and psychosocial constructs, none of which are included in our study, which focuses solely on machine-learning prediction of diabetes and hypertension using BDHS 2022 data. This appears to be an administrative mismatch of reviewer assignments. We respectfully request clarification or reassignment of this review to ensure appropriate evaluation of the manuscript’s content and methodology.

***Reviewer #2:***

**Comment:** The authors have provided a strong manuscript with clear research questions and robust methodology. I am satisfied with the clarity and presentation of the results. The manuscript has provided expected answers to the research questions raised.

**Response:** We thank the reviewer for the positive feedback and are pleased that the manuscript was found clear, methodologically sound, and responsive to the research questions. No additional revisions were requested.

***Reviewer #3:***

**Comment 1:** The authors applied six ML models but did not clearly justify why these specific algorithms were chosen over others. A brief rationale for each model’s relevance to epidemiological prediction would strengthen the methodological rigor.

**Response:** We thank the reviewer for this constructive suggestion. We have now clarified the rationale for selecting each machine learning algorithm, emphasizing their complementary strengths in epidemiological prediction, including handling nonlinearity, class imbalance, and high-dimensional categorical data.. We have added the following to the analysis section.

*“The six machine learning algorithms were selected to represent diverse and widely used modeling frameworks in epidemiological prediction. Random forest and gradient boosting methods are effective in capturing nonlinear relationships and interactions common in population health data. AdaBoost and XGBoost were included due to their robustness in handling imbalanced datasets and strong performance in health risk prediction. Support Vector Machine was selected for its ability to model complex decision boundaries in high-dimensional feature spaces, while Artificial Neural Networks were included to assess performance under flexible, nonparametric architectures. Together, these models allow a comprehensive comparison of predictive accuracy, discrimination, and interpretability relevant to public health applications.”*

**Comment 2:** While SMOTE and ADASYN were used, the rationale for applying both and their differential impact on model performance is not discussed.

**Response:** We appreciate this observation. We have expanded the Discussion section to clarify why both SMOTE and ADASYN were applied and how they differ conceptually. We have added the following to the Discussion section

*“Both SMOTE and ADASYN were applied to address outcome imbalance. SMOTE generates synthetic minority samples uniformly, whereas ADASYN adaptively generates samples near decision boundaries where misclassification risk is higher. Using both methods allowed us to assess robustness of model performance under different resampling strategies; results presented primarily reflect ADASYN due to its superior sensitivity in preliminary comparisons.”*

*“The use of ADASYN likely contributed to the high sensitivity observed across models by emphasizing difficult-to-classify minority cases. However, this may have occurred at the expense of specificity, underscoring the trade-off inherent in resampling strategies for low-prevalence outcomes.”*

**Comment 3:** The models show high sensitivity but very low specificity, indicating poor ability to correctly identify non-cases. The authors should discuss the public health implications and suggest ways to improve specificity.

**Response:** We agree and have expanded the Discussion to explicitly address the public health implications of high sensitivity and low specificity. We also outline potential methodological strategies to improve specificity in future work: *“High sensitivity with low specificity suggests that the models are effective for screening and identifying high-risk individuals but may overclassify non-cases. From a public health perspective, this trade-off may be acceptable in early detection programs where missing true cases is costlier than false positives. Future work could improve specificity by incorporating behavioral variables, continuous biomarkers, cost-sensitive learning, probability threshold optimization, or ensemble calibration techniques.”*

**Comment 4:** The study would benefit from a comparison with traditional statistical models.

**Response:** We thank the reviewer for this valuable suggestion. While a direct comparison was not conducted in this study, we now explicitly contextualize the added value of ML relative to traditional regression approaches: *“Compared with traditional regression models commonly used in epidemiology, machine learning approaches offer advantages in capturing nonlinear relationships and complex interactions without prespecified functional forms. Although logistic regression remains valuable for inference, the ML models used here demonstrated improved discrimination and ranking of predictors, highlighting their complementary role in population health analytics.”*

**Comment 5:** The abstract mentions “low specificity across models” but does not provide values.

**Response:** We have revised the Abstract to include representative specificity values for transparency: *“Sensitivity was high across models (up to 0.96 for diabetes and 0.90 for hypertension), while specificity remained low, particularly for diabetes prediction (as low as 0.13 in XGBoost).”*

**Comment 6:** BMI cutoffs for overweight/obesity should be specified.

**Response:** We agree and have explicitly stated the BMI cutoff used. *“Overweight/obesity was defined as body mass index (BMI) ≥25 kg/m².”*

**Comment 7:** The discussion could better highlight how ML findings translate to actionable public health interventions in Bangladesh.

**Response:** We have strengthened the Discussion to explicitly link ML findings to policy-relevant and actionable interventions. We added the following: *“These ML-derived risk profiles can inform targeted screening strategies within Bangladesh’s primary healthcare system, particularly for older adults, overweight individuals, and wealthier urban populations. Integration of such predictive tools into routine health surveys or electronic health systems could enable risk stratification and efficient allocation of limited preventive resources.”*

**Comment 8:** Check consistency in terminology (e.g., “AdaBoost” vs. “ADA Boost”).

**Response:** We have standardized terminology throughout the manuscript to “AdaBoost.”

**Comment 9:** The tenses in the work are not consistent.

**Response:** We have revised the manuscript to ensure consistent use of past tense for Methods and Results, and present tense for established knowledge and conclusions.

**Comment 10:** Ensure grammatical errors are corrected (e.g., “prevalce”).

**Response:** We conducted a thorough language and grammar review and corrected typographical and grammatical errors throughout the manuscript, including the noted example.

***Reviewer #4:***

**Comment 1:** *Tenses of reportage should be past in form; the manuscript contains present-tense statements such as “we aim to…”*

**Response:** We appreciate the reviewer’s observation. The manuscript has now been revised to ensure consistent use of **past tense** throughout all sections where study procedures or findings are described. The phrase “we aim to…” in the Introduction has been changed to past tense to align with scientific reporting conventions.

**Comment 2: A***void personalization (“We…”) and use passive voice such as “analysis was done.”*

**Response:** We thank the reviewer for this guidance. The manuscript has been revised to reduce first-person wording, particularly in Methods, Results, and Discussion. Passive, scientific constructions are now used (e.g., *“analyses were conducted,” “models were developed,” “comparisons were performed”*).

**Comment 3:** *Authors should compare findings to outcomes of similar studies from other settings.*

**Response:** We agree and have added comparisons with prior studies from Bangladesh and other LMICs, including those addressing diabetes, hypertension, clustering of NCD risk factors, and socioeconomic gradients. These contextual comparisons strengthen the interpretation of findings.

*“The observed associations align with findings from prior studies in Bangladesh and other LMICs, which similarly reported increasing diabetes and hypertension prevalence with age, higher socioeconomic status, and urban residence. Studies from India, Nepal, and Indonesia have also highlighted obesity, wealth, and urbanization as consistent predictors of cardiometabolic conditions, supporting the generalizability of these findings across South Asian populations.”*

**Comment 4:** *Use persuasive rather than obligatory tone in recommendations; avoid “should” in the Conclusion.*

**Response:** Thank you for the suggestion. The Conclusion has been revised to use a persuasive, non-directive tone. Mandatory wording such as “should guide” or “should prioritize” has been replaced with phrasing like “may help guide” and “may inform.”

**Comment 5:** *Was any measure taken to minimize confounding? If yes, include in manuscript to improve reproducibility.*

**Response:** We appreciate this thoughtful comment. Although the study used machine-learning models rather than conventional regression, several steps inherently reduced potential confounding:

1. **Inclusion of multiple demographic and socioeconomic variables** (age, sex, education, wealth quintile, residence, division) reduces omitted-variable bias.
2. **Bidirectional inclusion of diabetes and hypertension** accounted for mutual confounding effects.
3. **Cross-validation and resampling (ADASYN)** reduced overfitting, improving model validity.

We have clarified this in the Methods section.

*“Multiple demographic and socioeconomic variables were included to minimize potential confounding of associations among predictors and outcomes. The bidirectional inclusion of diabetes and hypertension also helped control for mutual confounding effects.”*

Dear Editor, we believe we were able to address all the comments. We are looking forward to your decision.

Sincerely,

Gulam Muhammed Al Kibria, MBBS, MSPH, PhD
