# Supplementary material for: Application of machine learning approaches to develop predictive models for diabetes and hypertension among Bangladeshi Adults
Source: PLOS Glob Public Health. 2026 Feb 17;6(2):e0004797. doi: 10.1371/journal.pgph.0004797 (PMC12912540; doi:10.1371/journal.pgph.0004797)
Supplement: Attachment — Submitted filename: response letter_2.docx [file pgph.0004797.s004.docx]

Gulam Muhammed Al Kibria, MBBS, MSPH, PhD

Johns Hopkins University Bloomberg School of Public Health

615 N Wolfe Street, Baltimore, MD 21205

**Academic Editor**

**PLoS Global Public Health**

Jan 27, 2026

Dear Editor:

This is to inform you that we have prepared a revised version of the manuscript ‘**Application of Machine Learning Approaches to Develop Predictive Models for Diabetes and Hypertension among Bangladeshi Adults’** for your consideration in the *PLoS* *Global Public Health*. We thank you and the reviewers for your time and effort. We have provided itemized responses below

**Comment 1:** Title: Please change Bangladesh adults to Bangladeshi adults.

***Response:*** *Edited, thanks for checking this!*

**Comment 2:** Use the term “sex” instead of “gender” throughout the manuscript.

***Response:*** *Edited this, thanks!*

**Comment 3:** Abstract: revise this sentence, particularly the first clause; it is difficult to understand for the lay audience. “Hypertension topped diabetes predictors, while overweight/obesity was the top predictor for hypertension, followed by age and diabetes.”

***Response:*** *Edited this sentence, thanks! (Current version: “Hypertension was the most common diabetes predictor, while overweight/obesity was the was the most common predictor for hypertension, followed by age and diabetes.”)*

**Comment 4:** Section “Potential Factors” in the methods: since the study included both males and females, why is only women’s age? So shouldn’t it be the age of men and women?

***Response:*** *Edited, thanks for checking this, it was a typo (current version:“age, sex, education level, household wealth quintile, overweight/obesity, and rural-urban place and division of residence”)*

Dear Editor, we believe we were able to address all the comments. We are looking forward to your decision.

Sincerely,

Gulam Muhammed Al Kibria, MBBS, MSPH, PhD
